# Dall-E in hand surgery: Exploring the utility of ChatGPT image generation

**DOI:** 10.1016/j.sopen.2025.04.012

**Published:** 2025-05-10

**Authors:** Daniel Soroudi, Daniel S. Rouhani, Alap Patel, Ryan Sadjadi, Reta Behnam-Hanona, Nicholas C. Oleck, Israel Falade, Merisa Piper, Scott L. Hansen

**Affiliations:** a University of California San Francisco, School of Medicine, San Francisco, CA, USA; b University of California San Francisco, Department of Surgery, Division of Plastic and Reconstructive Surgery, San Francisco, CA, USA; c Division of Plastic Surgery, Duke University Medical Center, Durham, NC, USA

## Abstract

**Background:**

Artificial intelligence (AI) has significantly influenced various medical fields, including plastic surgery. Large language model (LLM) chatbots such as ChatGPT and text-to-image tools like Dall-E and GPT-4o are gaining broader adoption. This study explores the capabilities and limitations of these tools in hand surgery, focusing on their application in patient and medical education.

**Methods:**

Utilizing Google Trends data, common search terms were identified and queried on ChatGPT-4.5 and ChatGPT-3.5 from the following categories: “Hand Anatomy”, “Hand Fracture”, “Hand Joint Injury”, “Hand Tumor”, and “Hand Dislocation”. Responses were graded on a 1–5 scale for accuracy and evaluated using the Flesch-Kincaid Grade Level, Patient Education Materials Assessment Tool (PEMAT), and DISCERN instrument. GPT 4o, DALL-E 3, and DALL-E 2 illustrated visual representations of selected ChatGPT responses in each category, which were further evaluated.

**Results:**

ChatGPT-4.5 achieved a DISCERN overall score of 3.80 ± 0.23. Its responses averaged 91.67 ± 0.29 for PEMAT understandability and 54.67 ± 0.55 for actionability. Accuracy was 4.47 ± 0.52, with a Flesch-Kincaid Grade Level of 9.26 ± 1.04. ChatGPT-4.5 consistently outperformed ChatGPT-3.5 across all evaluation metrics. For text-to-image generation, GPT-4o produced more accurate visuals compared to DALL-E 3 and DALL-E 2.

**Conclusions:**

This study highlights the strengths and limitations of ChatGPT-4.5 and GPT-4o in hand surgery education. While combining accurate text generation with image creation shows promise, these AI tools still need further refinement before widespread clinical adoption.

## Introduction

For more than a decade, artificial intelligence (AI) has significantly influenced medicine, offering groundbreaking advancements in clinical diagnostics and image analysis in fields such as radiology and histopathology [1]. The capabilities of AI extend into plastic surgery, where its applications are showing potential in predicting burn healing times, quantifying free-flap tissue perfusion, and measuring cranial differences in the diagnosis of craniosynostosis [[2], [3], [4]].

At the forefront of recent AI advancements are large language models (LLMs), sophisticated neural networks trained on vast text datasets capable of generating human-like language and responding contextually to user prompts [5]. A leading example among these LLMs is OpenAI's ChatGPT (San Francisco, CA), which has evolved rapidly from the launch of its initial free model, ChatGPT-3.5, in November 2022 to the recent release of its latest paid version, ChatGPT-4.5, in February 2025. Concurrently OpenAI has also released other models such as GPT-4o, GPTo1, GPTo3-mini, and GPTo3-mini-high. Other LLMs such as Google's Gemini (Mountain View, CA), and Claude, developed by Anthropic (San Francisco, CA) have also made significant advances in the field of LLMs [6]. Patients are increasingly using LLMs for medical information, with studies demonstrating their potential to improve accessibility and understanding [[7], [8], [9]]. However, concerns about the reliability and accuracy of these LLMs highlight the need for further evaluation of these educational tools [[10], [11], [12]].

Another prominent area of AI innovation is in text-to-image technology such as DALL-E, also developed by OpenAI. DALL-E translates descriptive textual inputs into corresponding visual representations using diffusion-based generative models [13]. Originally launched in January 2021, DALL-E significantly improved with the launch of DALL-E 2 in April 2022, followed by DALL-E 3 in September 2023, reflecting ongoing advancements in image clarity, detail, and accuracy. On March 25th, 2025 OpenAI released its new text to image generator known as the GPT-4o image generator which offers more accurate and photorealistic outputs. Despite these improvements, the application of AI-generated images to detailed and anatomically precise medical graphics remains challenging [[14], [15], [16]].

The use of AI in patient education, particularly LLMs and text-to-image generators, offers a new way to improve understanding of complex medical topics. In hand surgery, where clear communication of intricate anatomy and pathology is essential, these tools show strong potential [17]. Despite growing interest, no study has evaluated the accuracy and quality of combining LLM-generated text with AI-created visuals for patient education in this field. This study assesses the capabilities of ChatGPT-4.5 and GPT-4o image generation in creating educational materials on hand surgery. By comparing outputs from earlier models including ChatGPT-3.5, DALL-E 2, and DALL-E 3, we aim to explore how advancements in AI are shaping the evolution of patient education.

## Methods

In September 2023, questions associated with the most common search terms from a Google Trends analysis for hand surgery were queried using ChatGPT-4.5 with custom prompt engineering and ChatGPT-3.5 without any prompt modifications. The top three search terms were selected from the following categories: “Hand Anatomy”, “Hand Fracture”, “Hand Joint Injury”, “Hand Tumor”, and “Hand Dislocation”. New accounts were created on OpenAI's ChatGPT platform, and memory was disabled to prevent prior interactions from influencing responses.

For ChatGPT-4.5, customized prompts were designed to improve clarity, comprehensiveness, and accessibility (Table 1). These prompts were structured at a sixth-grade reading level following recommendations for patient education by the American Medical Association (AMA) and the National Institutes of Health (NIH) [18]. They included explicit role assignment (“As a medical expert”) to encourage structured responses, clear explanations with practical examples. Prompts also included instructions to cite sources. In contrast, ChatGPT-3.5 was queried using basic, unstructured prompts, mimicking common patient searches without modifications (Table 2).

**Table 1:** ChatGPT 4.5 inputs by topic with custom prompt engineering.

Hand anatomy	Hand fracture	Hand joint injury	Hand tumor	Hand dislocation
Anatomy of the Hand – As a medical expert, thoroughly explain the structure of the hand, including its bones, muscles, and joints. Describe how these parts work together to allow movement and function. Use clear and simple language suitable for patient education, aiming for approximately a sixth-grade reading level to ensure accessibility for diverse readers. Include practical examples of how the hand's anatomy helps people perform daily activities. Cite sources.	Hand Fracture – As a medical expert, thoroughly explain to a patient what a hand fracture is, how it occurs, and how it is treated. Use clear and simple language suitable for patient education, aiming for approximately a sixth-grade reading level to ensure accessibility for diverse readers. Describe treatment options, recovery expectations, and how patients can manage their healing process. Cite sources.	Metacarpophalangeal Joint Injury – As a medical expert, thoroughly explain to a patient what metacarpophalangeal joint injury is, what causes it, and how it is treated. Use clear and simple language suitable for patient education, aiming for approximately a sixth-grade reading level to ensure accessibility for diverse readers. Describe why it affects finger and hand movement, available treatments, and how patients can prevent complications during recovery. Cite sources	Hand Cancer – As a medical expert, thoroughly explain to a patient what hand cancer is, including possible causes, symptoms, and treatment options. Use clear and simple language suitable for patient education, aiming for approximately a sixth-grade reading level to ensure accessibility for diverse readers. Discuss diagnostic procedures to differentiate between benign and malignant growths. Cite sources.	Finger Dislocation – As a medical expert, thoroughly explain to a patient what a finger dislocation is, how it happens, and how it is treated. Use clear and simple language suitable for patient education, aiming for approximately a sixth-grade reading level to ensure accessibility for diverse readers. Include symptoms, medical treatment options, recovery guidance, and tips for preventing future dislocations. Cite sources
Wrist Anatomy – As a medical expert, thoroughly explain the anatomy of the wrist and why it is important. Describe the bones, joints, and muscles that make up the wrist and explain how they allow hand movement and support daily activities. Use clear and simple language suitable for patient education, aiming for approximately a sixth-grade reading level to ensure accessibility for diverse readers. Include practical examples of how the wrist functions in everyday life. Cite sources.	Metacarpal Fracture – As a medical expert, thoroughly explain to a patient what a metacarpal fracture is, how it occurs, and how it is treated. Use clear and simple language suitable for patient education, aiming for approximately a sixth-grade reading level to ensure accessibility for diverse readers. Describe treatment options, recovery expectations, and how patients can manage their healing process. Cite sources.	Mallet Finger – As a medical expert, thoroughly explain to a patient what mallet finger is, what causes it, and how it is treated. Use clear and simple language suitable for patient education, aiming for approximately a sixth-grade reading level to ensure accessibility for diverse readers. Describe why it affects finger movement, available treatments, and how patients can prevent complications during recovery. Cite sources.	Finger Tumor – As a medical expert, thoroughly explain to a patient what a finger tumor is, including possible causes, symptoms, and treatment options. Use clear and simple language suitable for patient education, aiming for approximately a sixth-grade reading level to ensure accessibility for diverse readers. Discuss how different types of tumors are diagnosed, when to seek medical attention, and how they may be managed. Cite sources.	Wrist Dislocation – As a medical expert, thoroughly explain to a patient what a wrist dislocation is, how it happens, and how it is treated. Use clear and simple language suitable for patient education, aiming for approximately a sixth-grade reading level to ensure accessibility for diverse readers. Include symptoms, medical treatment options, recovery guidance, and tips for preventing future dislocations. Cite sources
Hand Muscles Anatomy – As a medical expert, thoroughly explain all the different extrinsic and intrinsic muscles in the hand. Name each muscle, describe its function, and explain how each contributes to hand movement. Use clear and simple language suitable for patient education, aiming for approximately a sixth-grade reading level to ensure accessibility for diverse readers. Include practical examples of how these muscles help people perform daily activities. Cite sources.	Thumb Fracture – As a medical expert, thoroughly explain to a patient what a thumb fracture is, how it occurs, and how it is treated. Use clear and simple language suitable for patient education, aiming for approximately a sixth-grade reading level to ensure accessibility for diverse readers. Describe treatment options, recovery expectations, and how patients can manage their healing process. Cite sources.	Hand Joints – As a medical expert, thoroughly explain to a patient what hand joint injury is, what causes it, and how it is treated. Use clear and simple language suitable for patient education, aiming for approximately a sixth-grade reading level to ensure accessibility for diverse readers. Describe why it affects finger and hand movement, available treatments, and how patients can prevent complications during recovery. Cite sources.	Wrist Tumor – As a medical expert, thoroughly explain to a patient what a wrist tumor is, including possible causes, symptoms, and treatment options. Use clear and simple language suitable for patient education, aiming for approximately a sixth-grade reading level to ensure accessibility for diverse readers. Discuss how different types of tumors are diagnosed, when to seek medical attention, and how they may be managed. Cite sources.	Thump Dislocation – As a medical expert, thoroughly explain to a patient what a thumb dislocation is, how it happens, and how it is treated. Use clear and simple language suitable for patient education, aiming for approximately a sixth-grade reading level to ensure accessibility for diverse readers. Include symptoms, medical treatment options, recovery guidance, and tips for preventing future dislocations. Cite sources.

**Table 2:** ChatGPT 3.5 inputs by topic.

Hand anatomy	Hand fracture	Hand joint injury	Hand tumor	Hand dislocation
Anatomy of Hand	Hand Fracture	Metacarpophalangeal Joint Injury	Hand Cancer	Finger dislocation
Wrist Anatomy	Metacarpal Fracture	Mallet Finger	Finger Tumor	Wrist dislocation
Hand Muscles Anatomy	Thumb Fracture	Hand Joints	Wrist Tumor	Thumb Dislocation

Each ChatGPT response was analyzed using Flesch-Kincaid Grade Level to assess readability, which estimates the educational level required for comprehension [19]. The DISCERN instrument, a tool for assessing the quality and reliability of publications, was employed by two researchers (D.S and R.S) to evaluate all ChatGPT responses [20,21]. The DISCERN instrument utilizes a questionnaire with 16 items, each rated on a 5-point scale, to evaluate viewers' reactions to content-related questions [21]. A rating of 5 indicated ‘Yes,’ 3 indicated ‘Partially,’ and 1 indicated ‘No.’ DISCERN scores below 3 generally signify a poor quality source, while those above 3 indicate content with minimal drawbacks [22]. Additionally, the Patient Education Materials Assessment Tool (PEMAT) was employed by D.S and R.S to measure understandability and actionability, expressed as percentages, where higher scores indicated better usability for patient education [20]. Lastly, a surgeon (A.P) reviewed all responses for accuracy, scoring them on a 5-point Likert scale (1 = entirely incorrect, 5 = entirely correct).

To assess AI-generated visuals, GPT-4o, DALL-E 3, and DALL-E 2 were tasked with generating an image for one randomly selected ChatGPT response per category. These included Hand Muscle Anatomy, Metacarpal Fracture, Mallet Finger, Hand Cancer, and Thumb Dislocation. Since no standardized grading system exists for evaluating text-to-image anatomical accuracy, a qualitative assessment was conducted. Images were evaluated based on alignment with textual descriptions, presence of anatomical distortions, and accuracy of labeling.

The mean along with standard deviations of all metrics from ChatGPT outputs were calculated. A Chi-Square test was utilized to assess for differences between PEMAT understandability and actionability scores. A *p*-values less than 0.05 was considered statistically significant. Statistical Analyses were executed utilizing IBM SPSS Version 28.0 (IBM Corp., Armonk, N.Y.).

## Results

### Overall findings for ChatGPT-4.5 LLM outputs

Fifteen questions spanning five categories, Hand Anatomy, Hand Fracture, Hand Joint Injury, Hand Tumor, and Hand Dislocation, were presented to ChatGPT-4.5 (see Table 3, Table 4). The mean DISCERN reliability score was 3.61 ± 0.05, and the mean DISCERN quality score was 2.63 ± 0.16. The overall DISCERN score was 3.80 ± 0.23 out of 5. The mean PEMAT understandability score was 91.67 ± 0.29, and the mean PEMAT actionability score was 54.67 ± 0.55. The average accuracy rating was 4.47 ± 0.52 on a 5-point scale. The mean Flesch-Kincaid Grade Level was 9.26 ± 1.04, and the mean word count was 499.20 ± 63.70 words (Table 5).

**Table 3:** ChatGPT-4.5 LLM outputs.

Category	Prompt engineered input	ChatGPT-4.5 LLM output
Hand Anatomy	As a medical expert, thoroughly explain all the different extrinsic and intrinsic muscles in the hand. Name each muscle, describe its function, and explain how each contributes to hand movement. Use clear and simple language suitable for patient education, aiming for approximately a sixth-grade reading level to ensure accessibility for diverse readers. Include practical examples of how these muscles help people perform daily activities. Cite sources.	Our hands have two main types of muscles: extrinsic muscles (which start outside your hand) and intrinsic muscles (found entirely inside your hand). Each helps you move your hand and fingers in important ways. Extrinsic Muscles (starting in the forearm and ending in the hand) These muscles are responsible for powerful gripping, holding, and moving your fingers and wrist. 1. Flexor muscles (front side of the forearm) Flexor Digitorum Superficialis Function: Helps bend the middle joints of your fingers. Everyday Example: Holding a pencil tightly while writing or carrying grocery bags. Flexor Digitorum Profundus Function: Bends the tips of your fingers. Everyday Example: Buttoning your shirt or holding a needle. Flexor Pollicis Longus Function: Helps bend your thumb. Everyday Example: Texting, pressing remote buttons, or gripping objects tightly. 2. Extensor muscles (back side of the forearm) Extensor Digitorum Function: Straightens your fingers. Everyday Example: Opening your hand fully to wave. Extensor Pollicis Longus and Extensor Pollicis Brevis Function: Straighten and move your thumb away from your hand. Everyday Example: Giving a “thumbs-up.” Abductor Pollicis Longus Function: Moves your thumb away from your palm sideways. Everyday Example: Grabbing or picking up large objects like a cup or jar. Intrinsic Muscles (located entirely within the hand) These muscles control precise and delicate movements in your fingers and thumb. 1. Thenar Muscles (base of your thumb) Abductor Pollicis Brevis Function: Moves your thumb away from your palm. Everyday Example: Gripping and holding an apple. Flexor Pollicis Brevis Function: Helps your thumb bend at the base. Everyday Example: Pinching to hold small things like coins or paperclips. Opponens Pollicis Function: Moves your thumb toward your little finger (opposition). Everyday Example: Writing with a pen or picking up tiny objects. 2. Hypothenar Muscles (base of your little finger) Abductor Digiti Minimi Function: Moves your little finger away from your other fingers. Everyday Example: Grabbing wide objects like a glass. Flexor Digiti Minimi Brevis Function: Helps bend your little finger. Everyday Example: Grasping small handles or tools. Opponens Digiti Minimi Function: Helps your little finger move toward your thumb. Everyday Example: Making a tight grip around small items. 3. Lumbricals (tiny muscles between the fingers) Lumbricals Function: Allow fingers to bend at the base and stay straight at the tip, making a “table-top” shape. Everyday Example: Holding playing cards, typing on a keyboard, or holding a sandwich carefully. 4. Interosseous muscles (located between finger bones) Dorsal Interossei Function: Spread fingers apart. Everyday Example: Putting on gloves or spreading fingers to catch a ball. Palmar Interossei Function: Bring fingers back together. Everyday Example: Scooping up coins, beads, or other small items. 5. Adductor Pollicis (located near thumb) Adductor Pollicis Function: Pulls your thumb toward your palm and fingers. Everyday Example: Turning a key or holding a book firmly. How They Work Together: All these muscles work closely together. Extrinsic muscles handle bigger, stronger actions, while intrinsic muscles allow delicate, controlled tasks. When you pick up a cup, extrinsic muscles provide the power to grip it, and intrinsic muscles give your hand the exact shape needed for a stable hold. Sources: Drake, Richard, et al. Gray's Anatomy for Students. Elsevier, 2020. Tortora, Gerard J., and Derrickson, Bryan H. Principles of Anatomy and Physiology. Wiley, 2017. Netter, Frank *H. atlas* of Human Anatomy. Saunders Elsevier, 2023. This clear and simple overview will help you understand the muscles in your hand and how you use them every day!
Hand Fracture	As a medical expert, thoroughly explain to a patient what a metacarpal fracture is, how it occurs, and how it is treated. Use clear and simple language suitable for patient education, aiming for approximately a sixth-grade reading level to ensure accessibility for diverse readers. Describe treatment options, recovery expectations, and how patients can manage their healing process. Cite sources.	What is a Metacarpal Fracture? A metacarpal fracture is a broken bone in your hand. Your hand has five bones, called metacarpals, that connect your fingers to your wrist. When one or more of these bones break, it's called a metacarpal fracture. This type of injury often happens from a fall, hitting something with your hand, or during sports activities. How Does It Happen? Metacarpal fractures usually occur because: You fell onto your hand or wrist. You hit something hard with your closed fist (often called a “boxer's fracture”). Your hand was accidentally crushed or squeezed. When the bone breaks, you might hear or feel a snap or pop. Your hand can become swollen, bruised, and painful, and you might have trouble moving your fingers or making a fist. How Is It Treated? The treatment depends on how bad the fracture is. Here are the common treatments: 1. Splint or Cast For simple fractures, your doctor might put your hand in a splint or cast. This helps the bone heal by keeping it still. You'll usually wear the cast or splint for about 4 to 6 weeks. 2. Surgery For more serious fractures (when bones move out of place), you might need surgery. During surgery, your doctor might use pins, screws, or plates to hold your bones in place as they heal. What to Expect During Recovery Healing usually takes about 4 to 8 weeks, depending on your fracture. You may notice: Swelling and soreness slowly going away. Some stiffness when you first start moving your fingers again. Gradual return of strength and normal movement. Your doctor may recommend hand exercises or physical therapy to help you recover faster and better. How Can You Help Your Hand Heal? Here are tips to help you heal and get back to normal quickly: Keep your hand elevated above your heart to reduce swelling, especially in the first few days. Apply ice packs wrapped in a towel to your hand for 15–20 min several times a day to help with swelling and pain. Do exercises your doctor or therapist gives you. They help your hand stay strong and flexible. Avoid activities that put strain on your injured hand until your doctor says it's safe. When Should You See Your Doctor Again? Always keep your follow-up appointments. Call your doctor right away if: Your hand becomes more swollen, painful, or numb. Your cast or splint feels too tight. You notice changes in skin color or your fingers feel cold or numb. Sources: American Academy of Orthopaedic Surgeons (AAOS): Hand Fractures Cleveland Clinic: Metacarpal Fractures Johns Hopkins Medicine: Metacarpal Fracture Treatment This information will help you understand your injury and get better safely and quickly. Always ask your doctor or healthcare provider if you have any questions.
Hand Joint Injury	As a medical expert, thoroughly explain to a patient what mallet finger is, what causes it, and how it is treated. Use clear and simple language suitable for patient education, aiming for approximately a sixth-grade reading level to ensure accessibility for diverse readers. Describe why it affects finger movement, available treatments, and how patients can prevent complications during recovery. Cite sources.	Understanding Mallet Finger What is Mallet Finger? Mallet finger is a common injury that happens when the tendon (the strong cord that connects muscle to bone) at the tip of your finger is damaged. This injury makes it hard to straighten your fingertip completely. Your finger might stay bent down at the tip, making it look like a small hammer or “mallet,” which is why it's called “mallet finger.” How Does Mallet Finger Happen? Mallet finger usually happens when something hits your fingertip hard, like catching a ball incorrectly, bumping your finger into something, or hurting your finger during activities like sports or household chores. The impact damages or tears the tendon that normally helps straighten your finger. Sometimes, a small piece of bone may also break off with the tendon. Why Does Mallet Finger Affect Movement? Your finger moves because tendons connect your bones to your muscles. When the tendon at the tip of your finger tears or separates from the bone, it cannot pull your finger straight. That is why your finger stays bent down, even when you try to straighten it. Common Symptoms: You cannot straighten the fingertip on your own. The fingertip hangs down, bent. Pain, swelling, and bruising at the tip of the finger right after injury. How is Mallet Finger Treated? Treatment for mallet finger usually does not require surgery. Your doctor may recommend one or more of the following treatments: 1. Splinting (Most Common) Your finger will be placed in a small splint (plastic or metal brace) to keep your fingertip straight. You must wear this splint all the time for about 6 to 8 weeks. If you take it off early or too often, the tendon may not heal properly, and your finger could stay bent permanently. After these first weeks, your doctor may recommend wearing the splint part-time, usually at night, for several more weeks to ensure full healing. 2. Surgery (Less Common) Surgery is needed only if your injury is severe—like when a bone piece is broken off, or the injury does not heal with a splint. The surgery reattaches the tendon or bone piece and usually involves a small pin to hold things in place while healing. How to Prevent Complications During Recovery: Always wear your splint exactly as your doctor tells you. Keep your finger dry and clean to avoid skin irritation or infection. Do not bend your fingertip while it's healing; even a short break without the splint can undo healing. Attend follow-up visits with your doctor to make sure your finger heals correctly. After the Splint is Removed: Your finger might feel stiff or weak at first. Your doctor or therapist may give you gentle finger exercises to strengthen your finger and improve movement. Full healing can take several months, so be patient and follow instructions carefully. Sources: American Academy of Orthopaedic Surgeons (AAOS). (2021). Mallet Finger (Baseball Finger). Cleveland Clinic. (2022). Mallet Finger: Causes, Symptoms, Diagnosis & Treatment. Mayo Clinic. (2023). Mallet Finger: Symptoms and Causes.
Hand Tumor	As a medical expert, thoroughly explain to a patient what hand cancer is, including possible causes, symptoms, and treatment options. Use clear and simple language suitable for patient education, aiming for approximately a sixth-grade reading level to ensure accessibility for diverse readers. Discuss diagnostic procedures to differentiate between benign and malignant growths. Cite sources.	What is Hand Cancer? Hand cancer happens when abnormal cells grow out of control in your hand. Cancer in the hand is very rare, but it can affect bones, muscles, nerves, skin, or other tissues. Possible Causes Doctors don't always know exactly why someone gets hand cancer, but some things may increase the risk: Sun Exposure: Too much sunlight over many years can lead to skin cancer on your hand. Radiation Exposure: Past radiation treatments can sometimes cause cancer later on. Chemical Exposure: Contact with harmful chemicals over a long time. Family History: Cancer risk can sometimes run in families. Smoking: Smoking cigarettes can increase the risk of cancers anywhere in your body. (Source: American Cancer Society) Symptoms of Hand Cancer Symptoms you might notice include: A lump or bump on your hand that doesn't go away or keeps growing. Pain or soreness in one spot that won't heal. Skin changes, like sores or dark-colored patches. Swelling or stiffness in your fingers or wrist. Weakness or loss of feeling in your hand. How is Hand Cancer Diagnosed? Doctors will perform certain tests to find out if a lump or growth is cancer or harmless (benign): Physical Exam: The doctor examines your hand and checks how it moves and feels. Imaging Tests: X-rays, MRI, or CT scans take pictures of bones and tissues inside your hand. Biopsy: A small piece of tissue from your hand is taken and studied in a lab. This helps doctors know for sure if it's cancer or not. It's important to remember that many growths in the hand are not cancer (benign), such as cysts or harmless lumps. Only a doctor can tell you the difference. Treatment Options Treatment depends on the type, size, and stage of cancer: Surgery: The most common treatment. The doctor removes the cancerous lump or affected area. Radiation Therapy: High-energy rays destroy cancer cells. Sometimes used along with surgery. Chemotherapy: Medicine that kills cancer cells. Usually only needed if cancer has spread. Physical Therapy: Helps you regain movement, strength, and normal use of your hand after treatment. Early diagnosis helps the treatment work best. If you see something unusual or feel pain that won't go away, talk to your doctor right away. (Source: Mayo Clinic) When to See a Doctor Visit your doctor if you have a lump or growth on your hand that: Doesn't heal or keeps getting bigger. Feels painful or tender for several weeks. Changes color, shape, or texture. Your doctor can help determine if it's serious and start treatment early if needed. Remember, hand cancer is very rare, and many lumps on the hand are harmless. Always talk with your doctor about any concerns. Sources: American Cancer Society - Skin Cancer Mayo Clinic - Bone Cancer National Institutes of Health (NIH)
Hand Dislocation	As a medical expert, thoroughly explain to a patient what a thumb dislocation is, how it happens, and how it is treated. Use clear and simple language suitable for patient education, aiming for approximately a sixth-grade reading level to ensure accessibility for diverse readers. Include symptoms, medical treatment options, recovery guidance, and tips for preventing future dislocations. Cite sources.	Understanding Thumb Dislocation A thumb dislocation happens when the bones in your thumb are pushed out of their normal position. Usually, this involves the joint closest to the palm, called the metacarpophalangeal (MCP) joint, or the middle joint called the interphalangeal (IP) joint. Think of your thumb joints like hinges on a door—when a dislocation occurs, the “hinge” gets forced apart and can't move correctly. How Does a Thumb Dislocation Happen? Thumb dislocations usually happen because of an injury, like: Falling onto your hand with your thumb stretched out. Playing sports like basketball, football, or skiing, where your thumb can get pulled or bent too far. Any accident that jams or hits the thumb forcefully. Symptoms of Thumb Dislocation Common symptoms include: Pain right after the injury. Swelling around the thumb joint. Thumb may look crooked or bent. Difficulty moving or using the thumb. Sometimes, you may see a noticeable bump at the joint. If you experience these symptoms, you should see a doctor quickly. Do not try to fix your thumb yourself, as it could cause more damage. How Doctors Treat a Thumb Dislocation Here is what usually happens at the doctor's office: 1. Examination and Imaging The doctor will carefully examine your thumb. An X-ray is usually taken to check for broken bones and confirm the dislocation. 2. Putting the Joint Back into Place This process is called reduction. Your doctor will gently pull and push your thumb joint to slide the bones back into the right place. You may get medicine to numb your thumb or make you more comfortable. 3. Splint or Cast After the bones are back in place, your thumb will be kept still by using a splint or a cast. You might need to wear this for a few weeks, usually about 2 to 4 weeks, to help it heal properly. 4. Medication You might get medicine like ibuprofen (Advil, Motrin) to help with pain and swelling. 5. Physical Therapy or Exercises After the splint or cast is removed, you may need simple exercises or physical therapy to regain movement, strength, and flexibility in your thumb. In some cases, especially if there are torn ligaments (tissues connecting bones), you may need surgery. But most thumb dislocations heal without surgery. Recovery Guidance To help your thumb heal: Keep your thumb raised above your heart when resting to reduce swelling. Apply ice packs wrapped in a cloth for 15–20 min every 2–3 h during the first few days. Follow your doctor's instructions about wearing your splint or cast and taking medications. Complete any physical therapy exercises regularly. Your thumb should get back to normal use within about 4 to 6 weeks. But complete recovery might take longer if the injury was severe. Preventing Future Thumb Dislocations Here are tips to help you avoid hurting your thumb again: Wear protective gear (like gloves or braces) when playing sports that have a risk of thumb injury. Learn proper techniques when doing sports or activities (like skiing or basketball) to prevent falling or injuring your thumb. Do strengthening exercises regularly to keep your thumb muscles strong. Avoid putting too much stress on your thumb when gripping or carrying heavy objects. Sources: American Society for Surgery of the Hand: Thumb Dislocation Cleveland Clinic: Dislocated Finger MedlinePlus: Finger Dislocation Always talk with your healthcare provider for specific advice and instructions about your condition and recovery.

**Table 4:** ChatGPT-3.5 LLM outputs.

Category	Input	ChatGPT-3.5 LLM output
Hand Anatomy	Hand Muscles Anatomy	The muscles of the hand are complex and are responsible for the intricate and precise movements of the fingers and thumb. These muscles can be broadly categorized into two groups: extrinsic muscles and intrinsic muscles. Extrinsic Muscles: Extrinsic muscles of the hand originate outside of the hand itself, typically in the forearm, and their tendons extend into the hand to control finger and thumb movement. a. Flexor Muscles: Flexor Digitorum Profundus: This muscle flexes the distal interphalangeal (DIP) joints of the fingers. Flexor Digitorum Superficialis: This muscle flexes the proximal interphalangeal (PIP) joints of the fingers. b. Extensor Muscles: Extensor Digitorum: This muscle extends the fingers at the metacarpophalangeal (MCP), PIP, and DIP joints. Extensor Indicis: This muscle extends the index finger. Extensor Pollicis Longus: This muscle extends the thumb. Intrinsic Muscles: Intrinsic muscles of the hand are located within the hand itself, and they control fine motor movements of the fingers and thumb. They are responsible for various functions such as precision grip, power grip, and finger abduction/adduction. a. Thenar Muscles: Abductor Pollicis Brevis: Abducts the thumb away from the palm. Flexor Pollicis Brevis: Flexes the thumb. Opponens Pollicis: Helps to oppose the thumb to the other fingers for precision grip. b. Hypothenar Muscles: Abductor Digiti Minimi: Abducts the little finger. Flexor Digiti Minimi Brevis: Flexes the little finger. Opponens Digiti Minimi: Opposes the little finger. c. Interossei Muscles: Dorsal Interossei: Abduct the fingers away from the midline of the hand. Palmar Interossei: Adduct the fingers toward the midline of the hand. d. Lumbrical Muscles: These muscles flex at the MCP joints and extend at the PIP and DIP joints. They are important for fine control of finger movement. e. Adductor Pollicis: This muscle helps in adducting the thumb. These muscles work together to provide the hand with its incredible range of motion and dexterity. The proper functioning of these muscles is essential for various daily activities, including grasping objects, typing, writing, and more. Additionally, the tendons of these muscles are often interconnected, allowing for coordinated movements of the fingers and thumb.
Hand Fracture	Metacarpal Fracture	A metacarpal fracture refers to a break or fracture in one of the metacarpal bones of the hand. The metacarpals are the long bones that make up the palm of the hand and connect the wrist (carpal bones) to the bones of the fingers (phalanges). There are five metacarpal bones in each hand, and they are numbered from the thumb (first metacarpal) to the little finger (fifth metacarpal). Metacarpal fractures can result from various causes, including: Trauma: The most common cause of metacarpal fractures is direct trauma to the hand, such as a fall, impact, or sports-related injury. Crushing Injuries: These fractures can also occur in situations where the hand is crushed or caught in machinery or accidents. Repetitive Stress: Overuse or repetitive stress on the hand, particularly in activities that involve striking objects (e.g., boxing) or using hand tools, can lead to stress fractures in the metacarpals. The symptoms of a metacarpal fracture may include pain, swelling, bruising, deformity of the hand or fingers, and difficulty in moving or using the hand. The specific treatment for a metacarpal fracture depends on the severity and location of the fracture. Common treatment options include: Immobilization: For minor fractures, a splint or cast may be applied to immobilize the hand and allow the bone to heal. Closed Reduction: This is a procedure where a healthcare provider manipulates the broken bone fragments back into their proper position without surgery. Surgery: In more severe fractures or cases with significant displacement, surgery may be necessary. Surgical options include using pins, plates, screws, or wires to stabilize the fractured bone. Physical Therapy: After the initial healing process, physical therapy may be recommended to regain strength, range of motion, and functionality of the hand. The recovery time for a metacarpal fracture can vary depending on the severity of the injury and the chosen treatment method. Minor fractures may heal in a few weeks, while more complex fractures may take several months for a full recovery. It's essential to follow your healthcare provider's advice regarding post-fracture care and rehabilitation to ensure the best possible outcome and minimize the risk of complications. If you suspect a metacarpal fracture, seek medical attention promptly for an accurate diagnosis and appropriate treatment.
Hand Joint Injury	Mallet Finger	Mallet finger, also known as “baseball finger” or “drop finger,” is a condition that occurs when the extensor tendon at the end joint of a finger is damaged or torn. This typically happens due to a forceful impact or trauma to the fingertip, such as when trying to catch a ball or during a sporting activity. The extensor tendon is responsible for straightening the finger's last joint (the distal interphalangeal joint or DIP joint). When it is injured, the affected person loses the ability to straighten the finger fully at the DIP joint, and the finger remains in a flexed or bent position, resembling the shape of a mallet. This condition can be painful and affect the functionality of the finger. Treatment for mallet finger may include: Splinting: One of the most common treatments involves wearing a splint that immobilizes the DIP joint in a straightened position for several weeks. It is essential to wear the splint consistently to allow the tendon to heal properly. Surgery: In severe cases or when conservative treatments are not effective, surgical intervention may be necessary. Surgery typically involves repairing the damaged tendon or, in some cases, using pins or screws to stabilize the joint. Rehabilitation: After the initial treatment, rehabilitation exercises may be recommended to improve finger strength and flexibility gradually. It's crucial to seek medical attention if you suspect you have mallet finger, as untreated or improperly treated cases can lead to long-term deformity and loss of function in the affected finger. A healthcare professional, such as an orthopaedic specialist or hand surgeon, can assess the injury and recommend the most appropriate course of treatment based on its severity.
Hand Tumor	Hand Cancer	Cancer can affect various parts of the body, including the hands. However, it's important to clarify that “hand cancer” is not a specific type of cancer. Instead, cancer can develop in different tissues within the hand, just as it can occur in various organs and tissues throughout the body. Here are some types of cancer that can potentially affect the hands: Skin Cancer: Skin cancer can develop on any part of the body, including the hands. The most common types of skin cancer are basal cell carcinoma, squamous cell carcinoma, and melanoma. These often present as abnormal growths, moles, or sores on the skin. Soft Tissue Sarcoma: Sarcomas are cancers that develop in the soft tissues, which include muscles, tendons, and connective tissues. While they can occur anywhere in the body, they can also affect the hands and fingers. Bone Cancer: Bone cancer can affect the bones in the hand. Although bone cancer is relatively rare, it's possible for cancerous tumors to develop in the bones of the fingers or hand. Metastatic Cancer: Sometimes, cancer that originates in another part of the body can spread (metastasize) to the bones or soft tissues in the hand. This is known as metastatic cancer. If you suspect you have a growth, lump, or lesion on your hand that you are concerned about, it's essential to consult a healthcare professional. They can conduct a thorough examination, perform diagnostic tests, and determine the appropriate course of action, which may include biopsy, imaging studies, or other procedures to diagnose and treat any potential cancer. Remember that early detection and treatment are crucial for the successful management of cancer. If you have specific concerns about a particular type of cancer or its treatment, it's best to consult with a medical specialist who can provide you with personalized information and guidance.
Hand Dislocation	Thumb Dislocation	A thumb dislocation occurs when the bones that make up the thumb joint become separated from their normal position. The thumb joint is known as the metacarpophalangeal (MCP) joint, and it connects the thumb to the hand. Thumb dislocations can be painful and can affect hand function. Here's some information on thumb dislocations: Causes: Thumb dislocations can occur due to various reasons, including trauma or injury to the thumb. This can happen during sports activities, accidents, or falls where the thumb is forcefully bent or twisted. Types: There are different types of thumb dislocations: Dorsal Thumb Dislocation: This is the most common type, where the thumb dislocates toward the back of the hand. Palmar Thumb Dislocation: In this type, the thumb dislocates toward the palm side of the hand. Lateral Thumb Dislocation: This involves a dislocation to the side of the hand. Symptoms: Common symptoms of a thumb dislocation include severe pain, swelling, deformity, and loss of motion in the thumb. You may also experience numbness or tingling in the thumb or hand. Diagnosis: A healthcare provider will typically diagnose a thumb dislocation by examining the thumb, assessing its alignment, and ordering X-rays to confirm the dislocation and rule out any associated fractures. Treatment: The treatment for a thumb dislocation usually involves the following steps: Reduction: The healthcare provider will attempt to manually realign the dislocated bones, a procedure known as a reduction. This is often done under local anesthesia or sedation. Immobilization: After the reduction, the thumb is typically immobilized using a splint or a cast to allow the ligaments and tissues to heal. Rehabilitation: Physical therapy may be recommended to regain strength and mobility in the thumb. Medication: Pain relievers and anti-inflammatory medications may be prescribed to manage pain and inflammation. Complications: If not treated promptly and properly, thumb dislocations can lead to long-term issues, such as chronic instability, decreased range of motion, and arthritis in the thumb joint. It's essential to seek immediate medical attention if you suspect a thumb dislocation. Attempting to realign the thumb on your own can lead to further complications. A healthcare provider will assess the injury, determine the appropriate treatment, and provide guidance on rehabilitation to help you regain thumb function.

**Table 5:** Evaluation of ChatGPT4.5 with prompt custom engineering across hand surgery topics.

Measure	Hand anatomy	Hand fracture	Hand joint injury	Hand tumor	Hand dislocation	Overall score
DISCERN Reliability Score	3.51 ± 0.13	3.57 ± 0.00	3.60 ± 0.04	3.71 ± 0.07	3.64 ± 0.00	3.61 ± 0.05
DISCERN Quality Score	1.12 ± 0.21	2.83 ± 0.04	3.07 ± 0.31	3.05 ± 0.12	3.09 ± 0.11	2.63 ± 0.16
DISCERN Overall Score	3.50 ± 0.58	3.83 ± 0.29	3.67 ± 0.29	4.00 ± 0.00	4.00 ± 0.00	3.80 ± 0.23
PEMAT understandability score %	91.67 ± 0.29	91.67 ± 0.29	91.67 ± 0.29	91.67 ± 0.29	91.67 ± 0.29	91.67 ± 0.29
PEMAT actionability score %	53.33 ± 0.55	60.00 ± 0.55	60.00 ± 0.55	40.00 ± 0.55	60.00 ± 0.55	54.67 ± 0.55
Accuracy	4.67 ± 0.58	4.33 ± 0.58	5.00 ± 0.00	4.33 ± 0.58	4.00 ± 0.00	4.47 ± 0.52
Flesch-Kincaid Grade Level	9.39 ± 0.93	8.19 ± 1.64	9.55 ± 0.75	9.95 ± 1.49	9.23 ± 0.39	9.26 ± 1.04
Word count	587.00 ± 32.05	402.67 ± 68.54	473.00 ± 32.41	502.33 ± 110.60	531.00 ± 74.91	499.20 ± 63.70

### ChatGPT-4.5 LLM outputs by category

Hand Anatomy prompts received an overall DISCERN score of 3.50 ± 0.58, accuracy of 4.67 ± 0.58, PEMAT understandability of 91.67 % ± 0.29, actionability of 53.33 % ± 0.55, and a Flesch-Kincaid Grade Level of 9.39 ± 0.93. Hand Fracture prompts received an overall DISCERN score of 3.83 ± 0.29, accuracy of 4.00 ± 0.00, PEMAT understandability of 91.67 % ± 0.29, actionability of 60.00 % ± 0.55, and a Flesch-Kincaid Grade Level of 8.19 ± 1.64. Hand Joint Injury prompts received an overall DISCERN score of 3.67 ± 0.29, accuracy of 3.67 ± 0.58, PEMAT understandability of 91.67 % ± 0.29, actionability of 60.00 % ± 0.55, and a Flesch-Kincaid Grade Level of 9.55 ± 0.75. Hand Tumor prompts received an overall DISCERN score of 4.00 ± 0.00, accuracy of 5.00 ± 0.00, PEMAT understandability of 91.67 % ± 0.29, actionability of 40.00 % ± 0.55, and a Flesch-Kincaid Grade Level of 9.95 ± 1.49. Hand Dislocation prompts received an overall DISCERN score of 4.00 ± 0.00, accuracy of 4.67 ± 0.58, PEMAT understandability of 91.67 % ± 0.29, actionability of 60.00 % ± 0.55, and a Flesch-Kincaid Grade Level of 9.23 ± 0.39 (Table 5).

### Comparison to older models (ChatGPT-3.5)

The mean DISCERN reliability score was 2.51 ± 0.23, with a mean DISCERN quality score of 2.00 ± 0.93 and a combined DISCERN score of 3.27 ± 0.83. The average PEMAT understandability was 76.67 ± 6.17 %, and the actionability measure was 36.67 ± 20.40 %. Accuracy ratings averaged 4.40 ± 0.63 on a 5-point scale. The model's mean reading level was 11.04 ± 1.37. Evaluations specific to each category indicated higher accuracy (5.00 ± 0.00) in Hand Tumor responses compared to lower accuracy (3.67 ± 0.58) for Hand Joint Injury prompts; actionability measures ranged from 0.00 ± 0.00 % in certain subtopics (e.g., Hand Anatomy) to 60.00 ± 0.00 % in Hand Fracture queries, and Flesch-Kincaid Grade Levels varied from 9.60 ± 0.94 (Hand Anatomy) to 12.39 ± 1.28 (Hand Dislocation) (Table 6).

**Table 6:** Evaluation of ChatGPT3.5 responses across hand surgery topics.

Measure	Hand anatomy	Hand fracture	Hand joint injury	Hand tumor	Hand dislocation	Overall score
DISCERN Reliability Score	2.29 ± 0.00	2.57 ± 0.16	2.29 ± 0.00	2.76 ± 0.07	2.64 ± 0.23	2.51 ± 0.23
DISCERN Quality Score	1.05 ± 0.07	2.93 ± 0.08	2.05 ± 1.01	1.14 ± 0.16	2.86 ± 0.18	2.00 ± 0.93
DISCERN Overall Score	3.38 ± 0.98	3.67 ± 0.52	3.00 ± 0.89	2.67 ± 0.82	3.17 ± 0.41	3.27 ± 0.83
PEMAT understandability score %	77.27 ± 7.61	78.79 ± 4.69	74.24 ± 8.94	78.79 ± 4.69	74.24 ± 3.71	76.67 ± 6.17
PEMAT actionability score %	0.00 ± 0.00	60.00 ± 0.00	43.33 ± 8.16	40.00 ± 0.00	40.00 ± 0.00	36.67 ± 20.40
Accuracy	4.67 ± 0.58	4.00 ± 0.00	3.67 ± 0.58	5.00 ± 0.00	4.67 ± 0.58	4.40 ± 0.63
Flesch-Kincaid Grade Level	9.60 ± 0.94	10.94 ± 1.57	11.56 ± 1.23	10.73 ± 0.43	12.39 ± 1.28	11.04 ± 1.37
Word count	421.67 ± 67.28	375.33 ± 23.46	323.00 ± 66.43	319.67 ± 16.04	401.33 ± 34.12	368.20 ± 57.97

^a^PEMAT, Patient Education Materials Assessment Tool.

^b^Data shown as mean ± standard deviation.

## Text to image generation

### Hand muscle anatomy

**Fig. 1:**
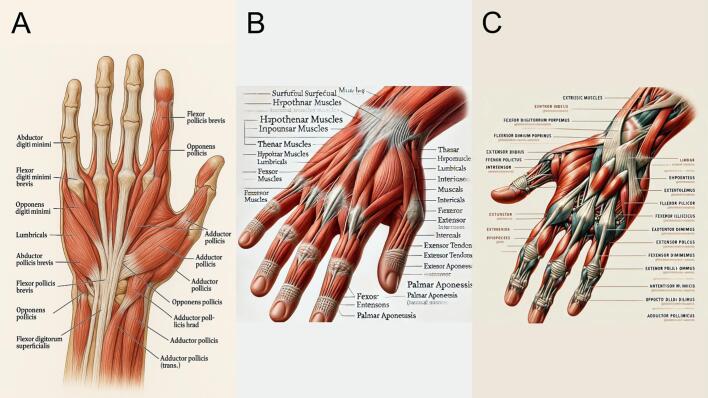
Hand muscle anatomy. (A) Generated using GPT-4o, (B) generated using DALL-E 3, and (C) generated using DALL-E 2.

GPT-4o's output (Fig. 1A) follows the prompt most closely, presenting a relatively accurate anatomical layout with correct digit count and some identifiable muscles such as the lumbricals and flexor pollicis brevis. However, there are multiple misspellings (e.g., “Opponens pollicis” repeated incorrectly), mislabeling of structures, and inaccuracies in tendon placement and orientation. DALL-E 3 (Fig. 1B) offers a more stylized and complex depiction with a variety of muscle-like structures, but it suffers from frequent anatomical errors, incorrect or invented muscle names, and inconsistent labeling. DALL-E 2 (Fig. 1C) is the least anatomically accurate: the labels are mostly illegible or nonsensical, the number of digits is incorrect, and although it resembles a hand, the representation is primarily artistic rather than educational. Among the three, GPT-4o demonstrates the highest adherence to the anatomical prompt but still falls short of medical accuracy.

### Metacarpal fracture

**Fig. 2:**
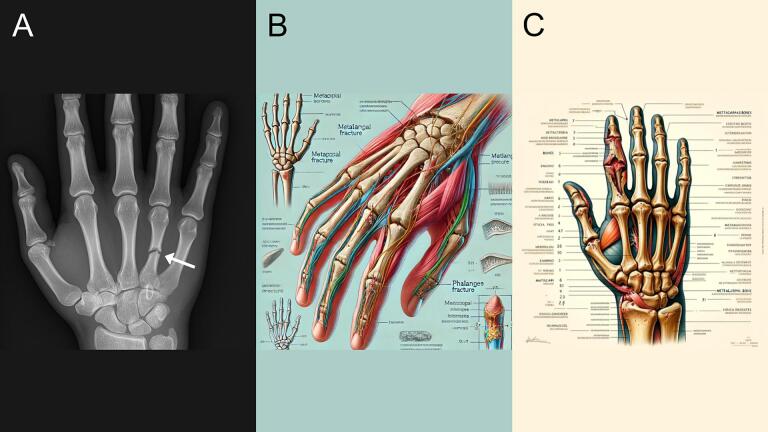
Metacarpal fracture. (A) Generated using GPT-4o, (B) generated using DALL-E 3, and (C) generated using DALL-E 2.

GPT-4o generated an X-ray-style image (Fig. 2A) accurately depicting a metacarpal fracture in the fourth metacarpal, clearly marked with an arrow. The image includes the correct number of metacarpals, middle phalanges, and an accurate amount of carpal bones. The distal radius and ulna are also present. The DALL-E 3 output (Fig. 2B) is a more artistic rendering. It lacks a clearly defined metacarpal fracture despite labeled indicators and includes surrounding vignettes with anatomical errors, such as incorrect labeling, spelling mistakes, and an inaccurate number of metacarpals and carpal bones. DALL-E 2's image (Fig. 2C) is the least anatomically reliable, featuring abstract and distorted bone shapes, particularly in the distal phalanges and carpal region. Labels are largely nonsensical or misspelled. Overall, GPT-4o followed the prompt with the greatest anatomical fidelity and educational value.

### Mallet finger

**Fig. 3:**
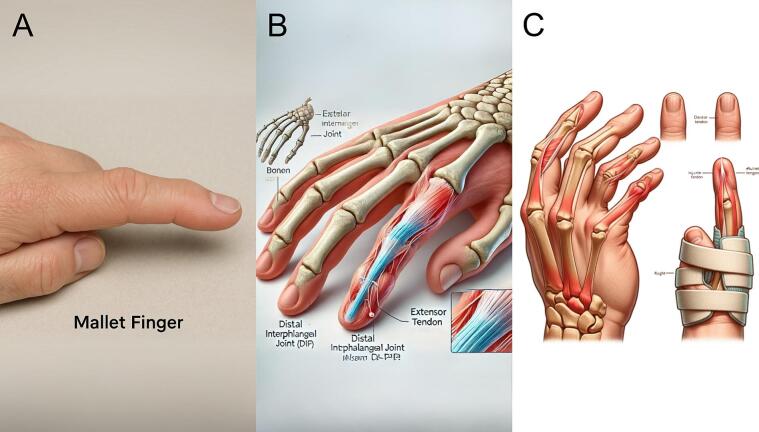
Mallet finger. (A) Generated using GPT-4o, (B) generated using DALL-E 3, and (C) Generated using DALL-E 2.

GPT-4o generated a photorealistic image of the second digit (Fig. 3A), showing slight flexion at the distal interphalangeal joint (DIP), consistent with mallet finger due to extensor tendon disruption. The image clearly conveys the typical flexion, though the extension lag could be more pronounced. It includes a correctly spelled “Mallet Finger” label, clearly identifying the condition. DALL-E 3's image (Fig. 3B) offers an artistic cross-section highlighting the extensor tendon insertion into the distal phalanx. However, it lacks visible rupture and contains anatomical inaccuracies, such as misspelled labels and incorrect bone counts. DALL-E 2's output (Fig. 3C) combines skeletal and soft tissue elements but lacks clarity and consistency. Its depiction of injury and splinting is undermined by distorted anatomy and inaccurate labels. Overall, GPT-4o and DALL-E 3 best captured the prompt, with GPT-4o offering the most clinically accurate portrayal.

### Hand cancer

**Fig. 4:**
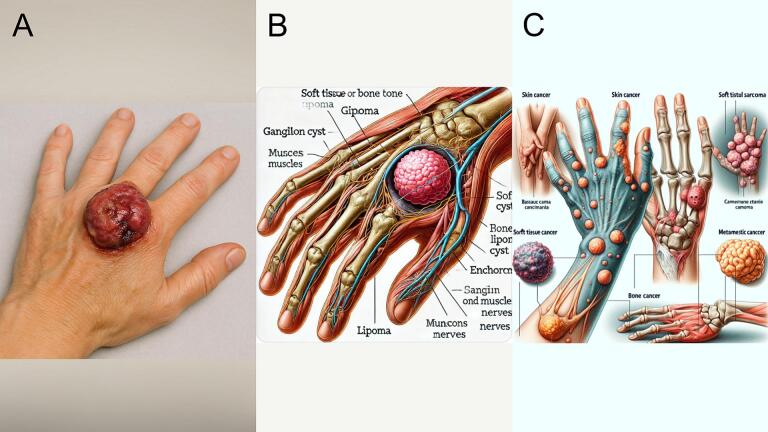
Hand cancer. (A) Generated using GPT-4o, (B) generated using DALL-E 3, and (C) Generated using DALL-E 2.

GPT-4o generated a photorealistic image of a hand with a large, irregular, reddish-purple lesion on the dorsal side near the metacarpals (Fig. 4A). The mass exhibits a glossy surface with ulcerated or necrotic areas, suggestive of malignancy, though the lack of overlying skin is anatomically inaccurate and dramatized. The image produced by DALL-E 3 (Fig. 4B) is an anatomical cutaway view showing a stylized tumor mass in the dorsal hand. The tumor is spherical and unrealistic in texture, with anatomical labels that are frequently misspelled or duplicated (e.g., “musces muscles,” “Gipoma”). DALL-E 2's output (Fig. 4C) is a cartoon-like and abstract compilation of various unrelated diagrams. Multiple tumor types are depicted in different locations, with no clear anatomical consistency, and the accompanying text contains numerous spelling errors and fabricated terminology (e.g., “camcimama ctanie camoma”). Overall, GPT-4o and DALL-E 3 adhered most closely to the prompt.

### Thumb dislocation

**Fig. 5:**
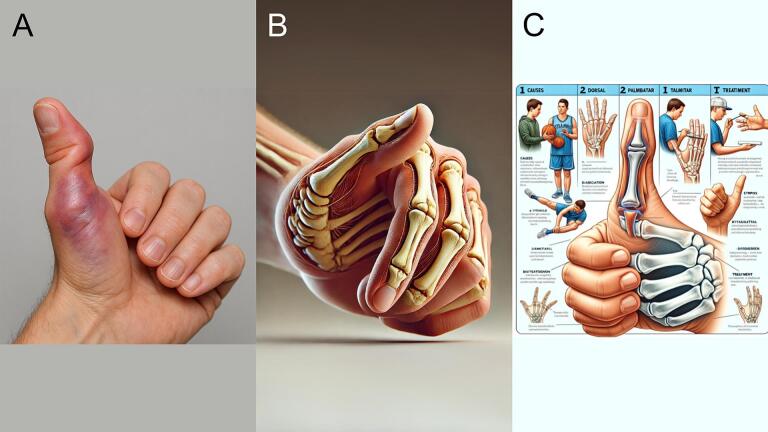
Thumb dislocation. (A) Generated using GPT-4o, (B) generated using DALL-E 3, and (C) generated using DALL-E 2.

GPT-4o generated a realistic image of a swollen, bruised thumb with deep purplish discoloration (Fig. 5A). The localized swelling, unnatural angulation, and joint prominence suggest trauma and joint misalignment, consistent with a dislocation, possibly at the interphalangeal (IP) joint. DALL-E 3 (Fig. 5B) produced an artistic anatomical rendering with visible bones and soft tissue, but the thumb's anatomy is inaccurate, and no clear dislocation is shown. DALL-E 2 (Fig. 5C) presents a cartoon-like infographic with distorted anatomy and multiple panels; while it gestures toward dislocation, the text contains spelling errors and the overall presentation lacks anatomical accuracy. GPT-4o followed the prompt most closely, providing the most clinically recognizable depiction.

## Discussion

In this study, we assessed how LLMs like ChatGPT-4.5 and ChatGPT-3.5, along with text-to-image generators GPT-4o, DALL-E 3, and DALL-E 2, perform in generating educational materials for hand surgery topics. Specifically, we analyzed the textual accuracy, reliability, readability, and actionability of AI-generated content, while also evaluating the anatomical precision and educational value of AI-produced images. Our findings reveal both the growing potential of these tools to enhance patient understanding and the limitations that must be addressed before widespread clinical adoption.

When guided by well-structured prompts, ChatGPT-4.5 consistently generated high-quality, reliable patient education content across a range of hand surgery topics, as reflected by high performance on the DISCERN assessment. High PEMAT understandability scores reflected that the content was easy for patients to comprehend. However, lower PEMAT actionability scores suggest that, while the material was understandable, it often lacked specific, patient-directed steps, limiting its usefulness in guiding real-world behavior. These findings align with the current literature on AI tools in clinical applications [23,24]. Prior studies have demonstrated that ChatGPT can accurately provide information about thumb arthritis but is limited in its ability to offer future treatment recommendations [23]. Another study evaluating ChatGPT-4's ability to answer questions regarding breast augmentation revealed that it could provide accurate information but struggled to generate patient-specific advice [4]. In our study, although ChatGPT-4.5 was prompted to produce text at a sixth-grade reading level, Flesch-Kincaid analysis revealed outputs at approximately a ninth-grade level, indicating a persistent gap between desired and actual readability. Outputs from ChatGPT-3.5 were included as an earlier baseline model, queried using unmodified prompts to reflect typical user interactions. Notably, the responses from ChatGPT-3.5 frequently lacked patient-centered guidance, produced less reliable and less understandable information, and exceeded recommended literacy levels, further highlighting the importance of both model selection and intentional prompt design in creating medical content.

Prompt engineering has been identified as a critical skill for medical professionals, with Meskó et al. emphasizing its role in enhancing the clarity, specificity, and educational value of LLM outputs [25]. Their recommendations, such as providing clinical context and assigning roles to LLMs, directly support our findings that tailored prompts significantly improved content quality in hand surgery education. A study by Yan et al. further supports this, showing that targeted prompt engineering was the key factor in improving the quality of AI-generated message drafts. As prompts were refined, the percentage of responses that physicians rated as ready to send or needing only minor edits rose from 62 % to 84 % (*p* < .001) [26]. This also aligns with similar studies published in the plastic surgery literature [[27], [28], [29], [30], [31]].

Our analysis of text-to-image outputs from DALL-E 2, DALL-E 3, and the recently released GPT-4o image generator underscores the rapid evolution of AI-driven medical illustration. DALL-E 2 introduced the concept of text-to-image generation but often produced abstract and artistic images lacking adequate anatomical detail. DALL-E 3 offered more refined visuals yet was prone to inconsistencies and frequent mislabeling. In contrast, GPT-4o emerged as the most advanced of the three models, typically yielding clearer, more anatomically correct depictions, such as a realistic X-Ray of a fractured metacarpal and a clinically recognizable mallet finger deformity. However, even GPT-4o faced difficulties in generating error-free anatomical details, especially for highly intricate structures like the muscle groups of the hand. This shortcoming is consistent with existing literature demonstrating that complex anatomy remains a challenging for AI-based image generation, given the precise labeling and spatial orientation required [[32], [33], [34], [35], [36]].

This study has several limitations. First, the number of prompts and hand surgery topics evaluated was limited, which may restrict the generalizability of our findings to other areas of medicine. Second, while ChatGPT-3.5 was included in the study, it was not intended as a direct comparator to ChatGPT-4.5. Rather, it served as a reference point to illustrate how LLM outputs have evolved over time. Because ChatGPT-3.5 responses were generated without prompt engineering, whereas ChatGPT-4.5 responses included structured prompts, direct comparison between the two is inherently limited and was not the primary aim of this study. Third, although structured tools like DISCERN and PEMAT allowed for grading of text-based outputs, no standardized evaluation framework currently exists for assessing the accuracy or educational value of AI-generated medical images. As a result, image evaluations were qualitative and subjective. Lastly, this study's accuracy ratings were performed by a single grader, which may have introduced individual bias despite clinical expertise.

Future research should prioritize the systematic evaluation and refinement of AI tools by gathering both quantitative and qualitative feedback from medical trainees and patients. Recognizing their distinct educational requirements, future studies should also investigate how to adapt AI outputs for specific user needs. Such adaptations might include altering the language complexity, content depth, and response length, thereby enabling exploration of AI tools' utility across diverse audiences. In addition, emerging AI capabilities, including text-to-video or image-to-video platforms such as OpenAI's “Sora”, present new opportunities to generate patient-specific multimedia content and expand the impact of AI in medical education and patient care.

## Conclusions

ChatGPT-4.5, when guided by structured prompts, demonstrated promising reliability, accuracy, and readability in creating patient education materials for hand surgery. GPT-4o image generation surpassed earlier models in producing more anatomically correct visuals, though inconsistencies and labeling errors persist. These findings underscore the importance of both model selection and prompt engineering for optimizing AI-generated content, while also revealing the need for improved standardization in evaluating text-to-image outputs. Despite its limitations, this rapidly evolving technology shows strong potential to enhance patient education, warranting broader exploration across diverse medical specialties.

## CRediT authorship contribution statement

**Daniel Soroudi:** Writing – review & editing, Writing – original draft, Visualization, Project administration, Methodology, Investigation, Formal analysis, Data curation, Conceptualization. **Alap Patel:** Writing – review & editing, Methodology, Conceptualization. **Ryan Sadjadi:** Writing – original draft, Data curation. **Reta Behnam-Hanona:** Writing – original draft, Data curation. **Nicholas C. Oleck:** Methodology, Conceptualization. **Israel Falade:** Writing – review & editing. **Merisa Piper:** Writing – review & editing. **Scott L. Hansen:** Writing – review & editing.

## Ethics approval and informed consent

Ethics approval and informed consent were not applicable as the study did not involve patient data or clinical trials.

## Declaration of Generative AI and AI-assisted technologies in the writing process

No generative AI or AI-assisted technologies were used in the writing of this manuscript.

## Funding sources

This research did not receive any specific grant from funding agencies in the public, commercial, or not-for-profit sectors.

Presented at: Plastic Surgery Research Council. May 16–19, 2024.

## Declaration of competing interest

The authors declare no competing interests or financial relationships that could influence the work reported in this manuscript.
